# Correction to “Spinal Cord Involvement in Neurosarcoidosis: A Case of Longitudinally Extensive Transverse Myelitis at Disease Onset”

**DOI:** 10.1002/ccr3.73211

**Published:** 2026-07-26

A. Tiwana , V. E. Nahra , and M. A. Hassan , “Spinal Cord Involvement in Neurosarcoidosis: A Case of Longitudinally Extensive Transverse Myelitis at Disease Onset,” Clinical Case Reports 14, no. 6 (2026): e72980, 10.1002/ccr3.72980.42328346 PMC13282162

The author order was incorrect and has been changed from Areeb Tiwana, Vicky Elias Nahra, Muhammad Adeel Hassan to Areeb Tiwana, Muhammad Adeel Hassan, Vicky Elias Nahra. Accordingly, the subsequent affiliations have been renumbered.

The online version of this article has been corrected accordingly.

We apologize for this error.

